# How Saccade Intrusions Affect Subsequent Motor and Oculomotor Actions

**DOI:** 10.3389/fnins.2016.00608

**Published:** 2017-01-12

**Authors:** Yasuo Terao, Hideki Fukuda, Shin-ichi Tokushige, Satomi Inomata-Terada, Yoshikazu Ugawa

**Affiliations:** ^1^Department of Neurology, University of TokyoTokyo, Japan; ^2^Department of Cell Physiology, Kyorin UniversityTokyo, Japan; ^3^Segawa Neurological Clinic for ChildrenTokyo, Japan; ^4^Department of Neurology, School of Medicine, Fukushima Medical UniversityFukushima, Japan; ^5^Fukushima Global Medical Science Center, Advanced Clinical Research Center, Fukushima Medical UniversityFukushima, Japan

**Keywords:** saccade intrusion, motor action, voluntary saccade, task switching, Parkinson's disease, eye-hand coordination

## Abstract

In daily activities, there is a close spatial and temporal coupling between eye and hand movements that enables human beings to perform actions smoothly and accurately. If this coupling is disrupted by inadvertent saccade intrusions, subsequent motor actions suffer from delays, and lack of coordination. To examine how saccade intrusions affect subsequent voluntary actions, we used two tasks that require subjects to make motor/oculomotor actions in response to a visual cue. One was the memory guided saccade (MGS) task, and the other the hand reaction time (RT) task. The MGS task required subjects to initiate a voluntary saccade to a memorized target location, which is indicated shortly before by a briefly presented cue. The RT task required subjects to release a button on detection of a visual target, while foveating on a central fixation point. In normal subjects of various ages, inadvertent saccade intrusions delayed subsequent voluntary motor, and oculomotor actions. We also studied patients with Parkinson's disease (PD), who are impaired not only in initiating voluntary saccades but also in suppressing unwanted reflexive saccades. Saccade intrusions also delayed hand RT in PD patients. However, MGS was affected by the saccade intrusion differently. Saccade intrusion did not delay MGS latency in PD patients who could perform MGS with a relatively normal latency. In contrast, in PD patients who were unable to initiate MGS within the normal time range, we observed slightly decreased MGS latency after saccade intrusions. What explains this paradoxical phenomenon? It is known that motor actions slow down when switching between controlled and automatic behavior. We discuss how the effect of saccade intrusions on subsequent voluntary motor/oculomotor actions may reflect a similar switching cost between automatic and controlled behavior and a cost for switching between different motor effectors. In contrast, PD patients were unable to initiate internally guided MGS in the absence of visual target and could perform only automatic visually guided saccades, and did not have to switch between automatic and controlled behavior. This lack of switching may explain the shortening of MGS latency by the saccade intrusion in PD patients.

## Introduction

Daily life requires an almost infinite number of actions that require eye-hand coordination (Engel and Soechting, 2003; Vercher et al., 2003; Crawford et al., 2004). For example, there is a close spatial and temporal coupling between the eyes and hand movements when subjects point to a peripheral target (Abrams et al., 1990; Helsen et al., 2000; Neggers and Bekkering, 2000, 2001; Ren et al., 2006). Similarly, in natural settings such as object manipulation, we first turn our gaze (central vision) to the object, and the hand subsequently reaches out to grasp it (Biguer et al., 1982; Prablanc and Martin, 1992).

This coordination of eye and hand movements has several advantages. First, by directing eye movements toward an object and foveating on it (i.e., placing it in the center of vision), the eyes provide spatial information for the hands (Crawford et al., 2004). Furthermore, pointing in general is more accurate when the gaze is fixed on the intended target, thereby avoiding the added processing of spatial updating for gaze shifts during pointing (Crawford et al., 2004). In some situations, gaze and arm movements appear to be guided by a common drive signal (Engel et al., 2000), and saccades are faster when accompanied by a coordinated arm movement (Epelboim et al., 1995; Snyder et al., 2002).

Therefore, it is reasonable to expect that if this coupling is disrupted by inadvertent saccade intrusions, subsequent motor actions would suffer from delays and lack of coordination. However, few studies have formally addressed this possibility. The most profound impact of saccade intrusions would be expected in complex action sequences performed in daily life. Such sequences involve a succession of individual object-related actions, each of which typically requires a turn toward an object, followed by fixation and finally manipulation monitored by vision (Land, 2009), where co-alignment of gaze and hand movements is ubiquitous (Land et al., 1999; Pelz et al., 2001; Pelz and Canosa, 2001). Multiple saccade intrusions during such sequences would seriously jeopardize this action sequence.

Insights into the disruption of organized eye-hand coordination would be beneficial in elucidating the pathophysiology of neurological patients with motor retardation and lack of coordination, who have a lot of saccade intrusions in daily actions.

In the present study, we investigated how saccade intrusions affect subsequent motor and oculomotor actions in two typical situations of daily action: when saccade intrusions precede or co-occur with oculomotor and motor actions. We used two tasks that require subjects to make motor/oculomotor responses upon the appearance of a visual signal. The memory guided saccade (MGS) task requires subjects to initiate a voluntary saccade to a memorized target location. The hand reaction time (RT) task requires subjects to release a button on detection of a visual target (cue).

Motor control and gaze control is known to be significantly affected by age (e.g., Munoz et al., 1998). In order to take into account age-related changes, we studied the performance of normal subjects of a variety of ages on the same tasks. We also studied patients with PD, who are impaired not only in initiating voluntary saccades but also in suppressing unwanted reflexive saccades. In previous studies (Leigh and Zee, 2006; Terao et al., 2011a, 2013c), we showed that PD patients are more impaired when initiating voluntary saccades and voluntary motor actions than during reflexive saccades and limb movements triggered by sensory cues. In contrast, PD patients are also impaired in suppressing inadvertent reflexive saccades toward a visual target or motor actions that are externally triggered (Rascol et al., 1989; Briand et al., 1999; Machado and Rafal, 2004; Chan et al., 2005; Joti et al., 2007; see Terao et al., 2013c for review).

We expected PD patients to show a larger effect of saccade intrusions on subsequent motor/oculomotor reactions, due to the shift in balance from reflexive to voluntary saccades. We hoped that by studying PD patients in this context, we would gain further insights into the effect of saccade intrusions on subsequent motor and oculomotor reactions.

## Subjects and methods

### Subjects

All experiments were conducted according to the declaration of Helsinki and were approved by the local ethical committee (School of Medicine, Tokyo university). For the main experiments to address how saccade intrusions affect the latency of subsequent voluntary saccades or motor actions, 86 normal elderly subjects (age: 55–80 years; mean ± standard deviation 65.8 ± 6.2 years) and 49 age-matched PD patients (age: 41–87 years; 70.1 ± 9.6 years; Hoehn-Yahr stages: I–IV) took part in the experiments after giving informed consent. All of the 86 normal subjects performed the MGS and RT tasks. In addition, in order to study how the effects of saccade intrusions varied with age, we also studied 415 normal subjects of various ages (age: 5–80 years), including the 86 elderly subjects recruited for the main experiment, also after obtaining written informed consent. The subjects comprised 70 subjects with ages between 5 and 14 years (group C), 79 subjects with ages between 15 and 24 years (group Y), 180 subjects with ages between 25 and 54 years (group M), and the 86 subjects with ages between 55 and 80 years (group E). In the PD group, 18 of 49 patients had recently been diagnosed with PD for the first time, and were not on any dopaminergic medication when they were studied. In the other patients, since discontinuation of the drugs was not possible for ethical reasons, the experiments were done at least 4 h after drug intake including L-DOPA based on our previous studies (Yugeta et al., 2008; Terao et al., 2011a) when there was only a small change in saccade performance for the oculomotor paradigms used.

### Experimental setup and behavioral paradigms

**Figure 1:**
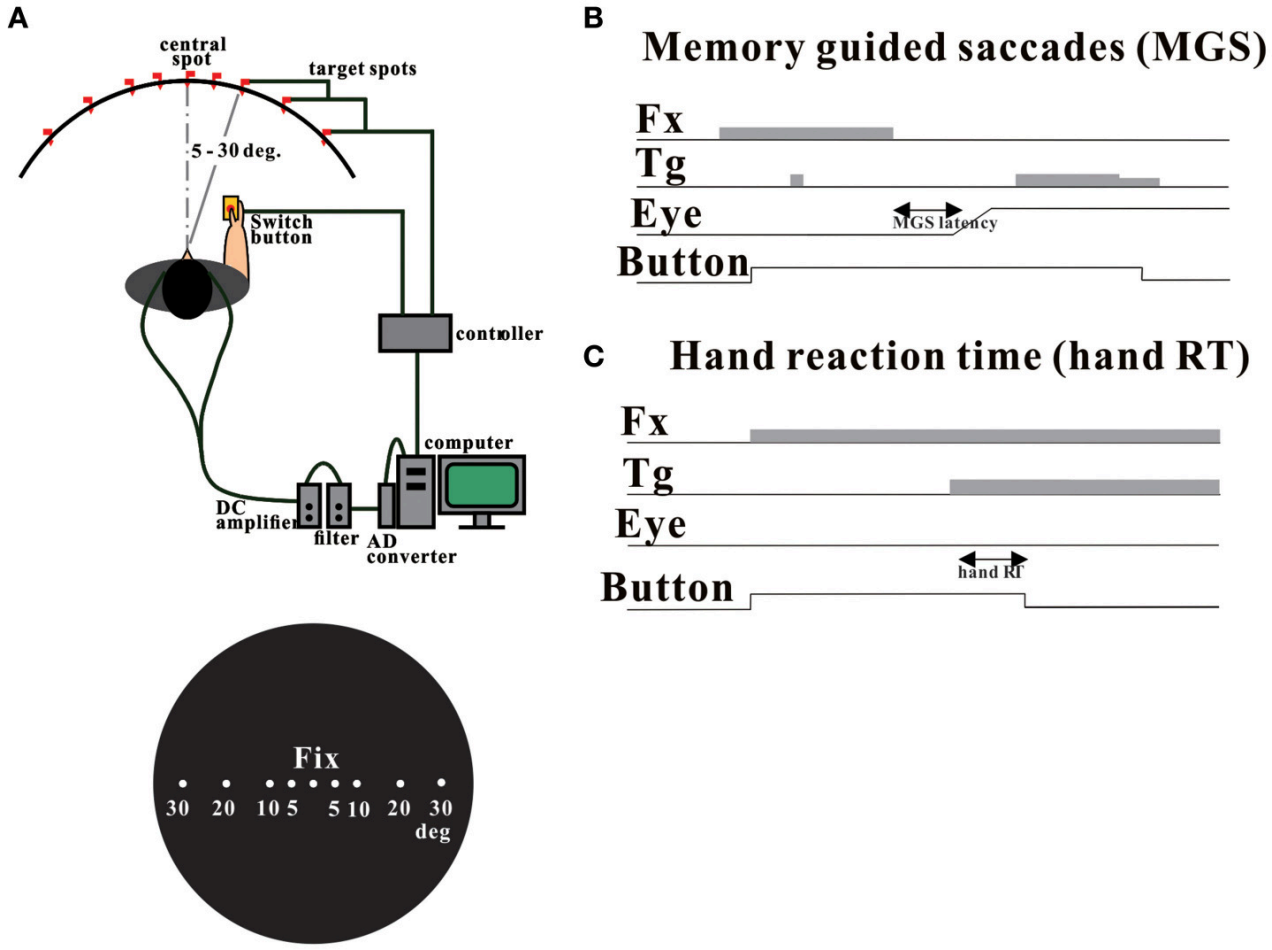
**Experimental setup (A)** and oculomotor tasks **(B**: MGS task, **C**: hand RT task). Adopted and modified from Terao et al. (2011a).

The experiments were performed in a dimly lit room with ambient light. On both sides of the dome, there were black shields to keep the light from directly coming in between the subjects' face and the dome. This setup was to ensure clear visibility of the targets, and at the same time to prevent the subjects from getting sleepy. As described previously (Kato and Hikosaka, 1992; Terao et al., 1998), subjects were seated in front of a black, concave, dome-shaped screen 90 cm in diameter, containing light-emitting diodes that served as the fixation point and saccade targets. Their heads were placed on a chin rest to restrain head movements (Kato et al., 1995; Terao et al., 1998; Figure 1A). They faced the center of the screen at a viewing distance of ~66 cm. The subjects held a microswitch button connected to a computer to control the task, allowing them to initiate and terminate the tasks (see below) by pressing the button with one of their thumbs.

Horizontal electro-oculographic (EOG) recordings were made with two Ag-AgCl gel electrodes placed at the bilateral outer canthi, and vertical EOG recordings were recorded by electrodes placed just above the upper lid and below the lower lid as described previously (Terao et al., 1998, 2011a,b, 2013a,b,c, 2016a,b). The signals were fed to a DC-amplifier (AN-601G; Nihon-Kohden, Tokyo, Japan), low-pass filtered at 20 Hz, and then digitized (500 Hz). Eye movement calibration took place before each test session. A target appeared 20 degrees to the left and right of the fixation point. While the subjects fixated this spot, we adjusted the gain of EOG so that the current eye position displayed on the computer monitor matched the target position displayed on the screen. Thus calibrated, EOG is roughly linear to 30 degrees, with a resolution of 0.5°. Our method has been shown to achieve a good correlation with recordings obtained via a video-based eye tracking system that is now widely used for recording saccades (Eyelink II; SR Research, Kanata, Ontario, Canada) and has been successfully used in a number of published studies (Terao et al., 1998, 2007, 2011a,b, 2013a,b,c, 2016a,b; Okano et al., 2010).

The subjects performed both the MGS task and the hand RT task.

In the MGS task (Figure 1B), while the subject fixated the central spot, a peripheral stimulus (“cue”) appeared briefly for a period of 50 ms. The subjects were required to maintain fixation until the fixation point was turned off (delay period), when the subjects had to make a saccade based on their memory to the spatial location where the cue had appeared. Thus, the imperative signal for response was the extinction of the central fixation point in this task. The time interval between the cue presentation and the extinction of fixation point was randomly varied across trials between 1.6 and 2.4 s (6 levels: 1600, 1760, 1920, 2080, 2240, 2400 ms). The target spot was turned on for a second time at 600 ms after the offset of the fixation point, so that the subjects could confirm the exact location of the target and correct their gaze positions. We measured the latency of saccades from the time of extinction of the central fixation point. Fifty trials each were implemented for the MGS task.

In the hand RT task (Figure 1C), a central spot of light came on shortly after the subject pressed a button, and stayed on throughout each trial, and the subjects were required to keep fixating on this point. Thereafter, another spot came on at various eccentricities and the subjects released the button as soon as possible while fixating the central cross. Thus, the imperative signal for response was the presentation of the peripheral visual target. The reaction time (RT) of button release was also measured from the time of target presentation. The time interval between the fixation point onset and the appearance of the target (fixation point duration) was randomly varied across trials between 1.5 and 3.0 s (6 levels: 1500, 1800, 2100, 2400, 2700, 3000 ms). In the hand RT task, RT was measured from the time of target presentation to the time of button release. Forty trials each were administered for the MGS task.

Both MGS and RT tasks required the subjects to keep gazing at the central fixation point until an imperative signal allowed them to initiate a quick voluntary oculomotor or motor response. Saccades unintentionally made to the cue during the delay period of MGS task were termed *saccades to cue*. Inadvertent saccades made to the target in the RT task were termed *saccades to target*.

### Data analysis and statistical assessment

Four parameters were determined off-line for each saccade: onset latency, amplitude, duration, and peak velocity. The onset of an eye movement was defined as the time when velocity and acceleration exceeded predetermined values (28°/s and 90°/s^2^). Eye movement was accepted as a saccade based on its velocity and duration. After the onset, the velocity had to exceed 88°/s, and this suprathreshold velocity had to be maintained for at least 10 ms. The end of an eye movement was determined where the velocity decreased below 40°/s. The total duration had to be more than 30 ms. Records contaminated by noise were excluded from the analysis as well as those with onset latency <100 ms.

In the MGS and RT tasks, we studied whether saccade intrusions (i.e., saccades to cue or saccades to target) made just before a voluntary eye movement or a voluntary motor action affect the latency of these actions. We investigated how saccades to cue affect the latency of MGS subsequently performed and how saccades to target affect the RT of button release in the hand RT task. For this purpose, we calculated the mean MGS latency for each subject, separately in trials in which the subjects made a saccade to cue and in which they did not. Then, we compared the latencies of MGS between these two types of trials, using the paired Student's *t*-test. After pooling data for all trials in all subjects, a frequency histogram of MGS latency was constructed for both types of trials, separately for normal control subjects and PD patients. The average of MGS latency was calculated for each type of trial in each subject and was compared between trials with saccades to cue and those without using the paired Student's *t*-test. A *p* < 0.05 was considered statistically significant.

We next looked at how the effect of saccade intrusion on MGS latency and hand RT varied with different ages. Similarly to the analyses above, frequency histograms of MGS latency and hand RT were constructed for trials with or without saccade intrusion, separately for subjects of the four age ranges (5–14, 15–24, 25–54, 55–80 years).

Statistical analyses were conducted using SPSS 10 (SPSS Japan, Tokyo, Japan). In each individual subject, the mean MGS latency and hand RT time was also calculated for trials with or without saccade intrusion. These were subjected to repeated measures analysis of variance (ANOVA) with saccade intrusion (two levels: presence or absence of saccade intrusions) as a within-subject factor and age range (four levels: 5–14, 15–24, 25–54, 55–80 years) as a between-subject factor to see how age affected the effect of saccade intrusions on subsequent voluntary saccades or motor actions. Since there were no significant difference between directions (effect of direction: *p* = 0.3211), the results for the two directions were put together. The paired Student's *t*-test was conducted to compare MGS latency between trials with and without saccades to cue, and to compare hand RT between trials with and without saccades to target in PD patients on an individual subject basis.

## Results

### The effect of saccade intrusion on the latency of subsequent voluntary saccades (MGS task)

**Figure 2:**
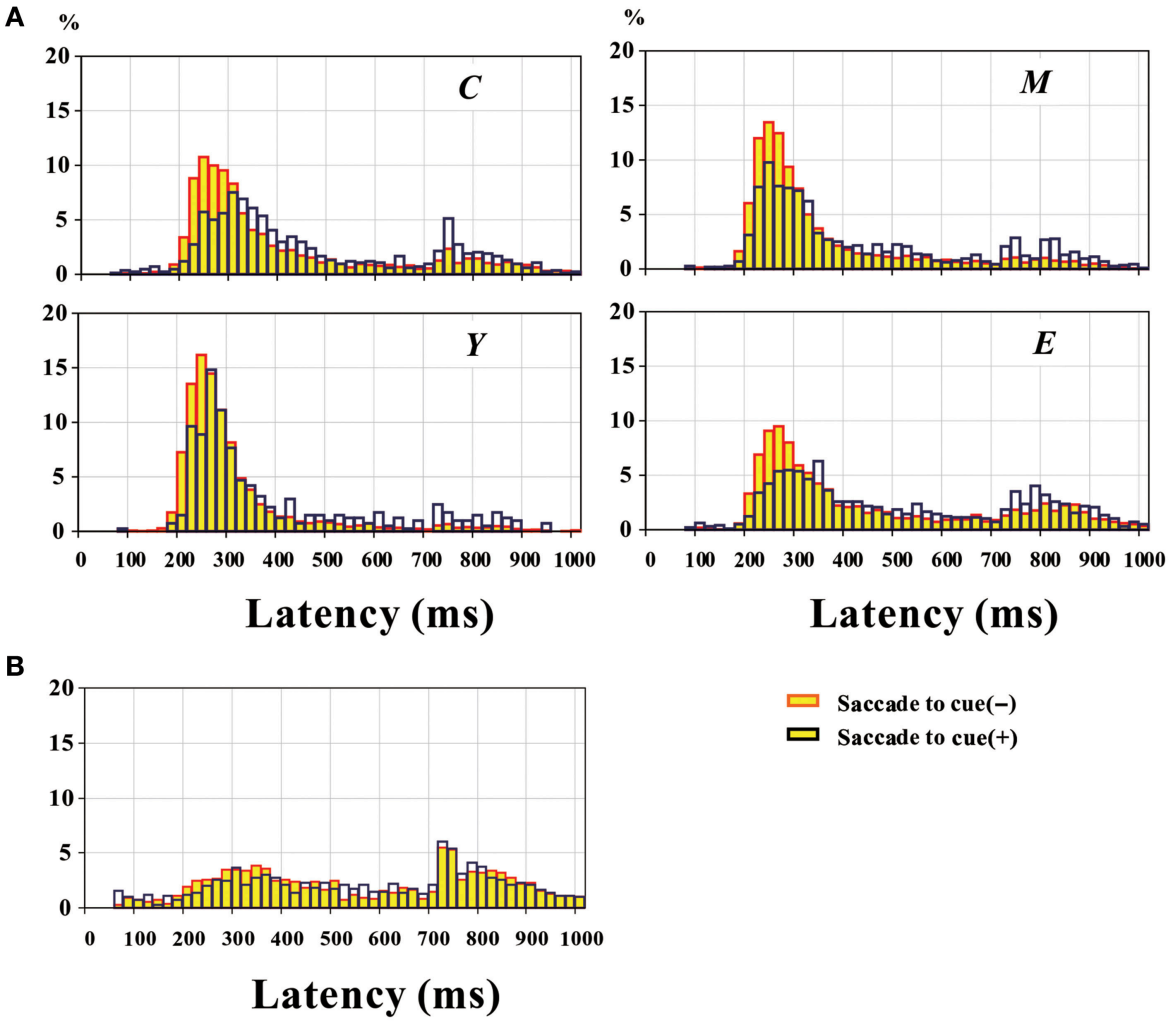
**Distribution of MGS latency pooled across all participants, in normal subjects (A)** and PD patients **(B)**. The yellow bars represent trials without saccades to cue, and blue bars those with saccades to cue. Plots for normal subjects **(A)** are given separately for the four groups of different age ranges. C: 5–14 years; Y: 15–24 years; M: 25–54 years; E: 55–80 years.

In the MGS task, normal subjects made saccades to cue in 24.8 ± 1.8% of the trials. Figure 2A shows the distribution of saccade latency in normal subjects, both in trials in which the subjects made saccades to cue (blue bars) and in which they did not (yellow bars), separately for the four age groups (C: 5–14 years; Y: 15–24 years; M: 25–54 years; E: 55–80 years). In all age groups, the histograms show that the latency of subsequent voluntary saccades was increased when they made a saccade to cue.

Each of the histograms shows two peaks. Based on the observations made during the task performance, the earlier peak was considered to correspond to a correctly performed MGS (in the absence of a visual target), whereas the second peak was considered to correspond to visually guided saccades made in response to the target presented for the second time in the MGS (600 ms after offset of the central fixation point). Based on the histogram, we set a cutoff value of 670 ms; this cutoff value was based on the overall distribution of correctly performed MGS latency for normal subjects at each age range and that of visually guided saccades to the target presented for the second time in the MGS task for the same subjects. In order to maximally separate out these two distributions, we set the cutoff value where these two distributions meet (670 ms). Saccades with latencies under this cutoff were considered correctly performed MGS, whereas saccades with latencies above the cutoff were considered visually triggered saccades to the target presented for the second time. The first peak was smaller and the second peak larger when saccades to cue were made than when they were not made. As shown in Figure 2A, the proportion of trials with a latency above 670 ms was larger in trials with saccades to cue than in trials without (proportion of trials with latency>670 ms: C: without saccades to cue: 15.2%, with saccades to cue: 24.1%; Y without saccades to cue: 4.6%, with saccades to cue: 13.8%; M without saccades to cue: 9.2%, with saccades to cue: 20.4%; E without saccades to cue: 22.5%, with saccades to cue: 31.4%).

In normal subjects, we compared the mean MGS latencies of each individual subject in trials with and without saccades to cue. MGS latency with saccades to cue (465.7 ± 7.7 ms) was significantly longer than that without saccades to cue [386.0 ± 6.2 ms; main effect of saccades to cue: *F*_(1, 392)_ = 96.013, *p* < 0.0001]. The difference in MGS latencies between trials in which the subject made saccades to cue and those in which the subject did not was significant across all age range groups, although the magnitude of the increase in latency was smallest in the youngest subject group [Y: 5–14 years; effect of age group: *F*_(3, 1176)_ = 96.013, *p* < 0.0001; saccade to cue X age group: *F*_(3, 1176)_ = 2.889; *p* = 0.0354; *post-hoc* analysis: group C: *p* = 0.0484; Y: *p* = 0.0003; M: *p* < 0.0001; E: *p* < 0.0001; group C: 30.2 ± 18.9 ms, Y: 64.2 ± 14.6 ms, M: 52.7 ± 12.2 ms, E: 60.4 ± 21.1 ms].

PD patients made significantly more saccades to cue in 47.2 ± 3.4% of the total trials on average, as compared to normal subjects (24.8 ± 1.8%; difference: Student's *t*-test: *p* < 0.0001). In contrast to normal subjects, overall, PD patients could initiate saccades slightly faster after they have made a saccade to cue (583.0 ± 18.8 ms) than when they did not (621.1 ± 20.9 ms; paired Student's *t*-test: *p* = 0.037; Figure 2B).

Similarly to normal subjects, we noted that the latency distribution comprised two distinct peaks. Pooling all trials across all subjects, the first and second peaks of the latency distribution comprised 56.5 and 43.5% of all trials without saccades to cue, whereas they comprised 54.8 and 45.2% of all trials with saccades to cue. Thus, unlike in normal subjects, the latency distribution did not show an evident difference between trials with and without saccades to cue, despite the slight decrease in latency on average in trials with saccades to cue, as shown above (chi-square test: *p* = 0.4653).

**Figure 3:**
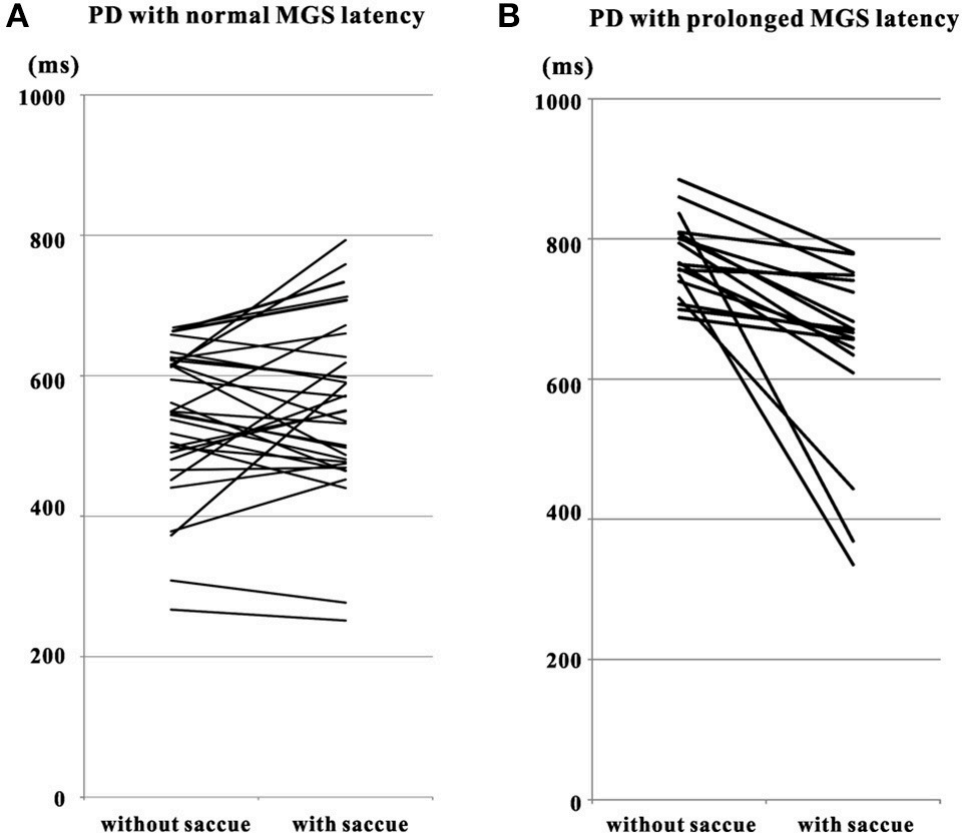
**Comparison of MGS latency of PD patients in trials with and without saccades to cue**. Plots are constructed separately for PD patients with MGS latency within the normal range **(A)** and those with prolonged MGS latency **(B**, with MGS latency > 670 ms). Data from the same patients are connected for trials without saccades to cue (left side of each figure) and with saccades to cue (right side of each figure). Note that short MGS latencies tend to become prolonged after saccades to cue **(A)**, whereas longer MGS latencies tend to get shorter **(B)**. Saccue: saccades to cue.

Looking closer into the saccade latency of individual patients, 30 PD patients could perform MGS with a normal latency (under 670 ms) without saccades to cue (Figure 3). Including both trials with and without saccades to cue, these patients could perform MGS correctly in as many as 52.6 ± 3.6% of the trials within the time limit of 670 ms, although the success rate was significantly lower than the normal success rate in age-matched control subjects (69.5 ± 2.2%, *p* = 0.00018). In these patients, the latency of saccades was 532.8 ± 18.5 ms without saccades to cue and 548.4 ± 21.8 ms with saccades to cue.

In 17 out of these 30 patients, MGS latency was greater with saccades to cue than without. In the remaining 13 patients, however, there was a small decrease in MGS latency with saccades to cue, by an amount comparable to or less than the standard deviation of MGS latency (157.5 ± 34.4 ms) in all but one of the patients. Overall, the difference in MGS latency between trials with and without saccades to cue did not reach significance (paired Student's *t*-test: *p* = 0.293).

In contrast, 19 PD patients showed a mean MGS latency above 670 ms even without saccades to cue. Including trials with and without saccades to cue, these patients were unable to correctly perform MGS within the cutoff time limit in most trials. In these patients, the MGS success rate was 23.8 ± 3.7%, which was significantly lower than in the 29 patients with normal mean MGS (*p* < 0.0001). These PD patients tended to make visually triggered saccades in response to the second target presentation. With saccades to cue, all of these patients performed saccades with a significantly shorter latency (642.5 ± 30.8 ms; paired Student's *t*-test: *p* = 0.000198) than in trials without saccades to cue (773.9 ± 12.9 ms).

Elderly normal subjects with normal MGS latency showed a significantly longer MGS latency (514.0 ± 15.7 ms; paired Student's *t*-test: *p* < 0.0001) in trials with saccades to cue than in trials without (423.1 ± 11.9 ms). In contrast, elderly normal subjects with prolonged MGS latency performed saccades with a significantly shorter latency (635.0 ± 23.8 ms; paired Student's *t*-test: *p* < 0.0001) in trials with saccades to cue than in trials without saccades to cue (747.7 ± 16.0 ms). Therefore, the elderly normal subjects with prolonged MGS latency behaved similarly to the PD patients with prolonged MGS latency, i.e., significantly shorter MGS latency in trials with saccades to cue than without. In contrast, elderly normal subjects with normal MGS latency showed longer MGS latency in trials with saccades to cue than without, unlike PD patients with normal MGS latency who showed comparable MGS latency in trials with and without saccades to cue.

Thus, PD patients whose MGS latency was longer than the normal range performed the task faster after making saccades to cue, similarly to normal subjects with prolonged MGS latency. In contrast, in PD patients who could perform MGS with a relatively normal latency, saccade intrusion did not largely change the initiation of subsequent voluntary saccade, unlike normal subjects with normal MGS latency in whom MGS latency was shorter in trials without saccades to cue than in those with saccades to cue.

### The effect of saccade intrusion on the initiation of subsequent motor reaction (hand RT task)

**Figure 4:**
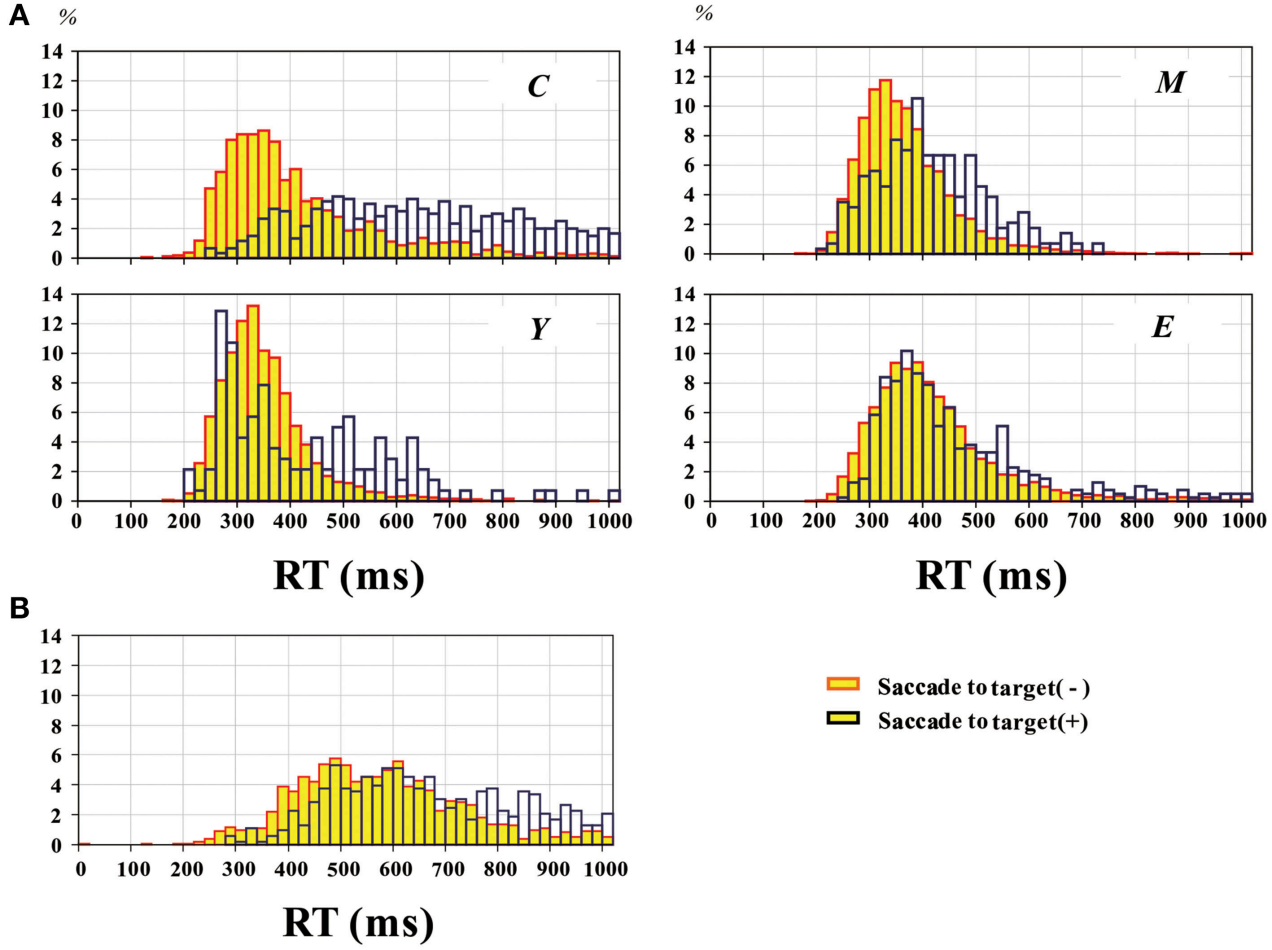
**Distribution of RT pooled across all participants, in normal subjects (A)** and PD patients **(B)**. The yellow bars represent trials without saccades to target, and blue bars those with saccades to target. Plots for normal subjects are given separately for the four groups of different age ranges as in Figure 2.

In normal subjects, saccade intrusion (saccades to target) delayed the hand RT (Figure 4A). Mean hand RT was significantly longer with saccades to target (376.1 ± 14.6 ms; blue bars) than without [yellow bars; 282.7 ± 5.8 ms; effect of saccades to target: *F*_(1, 190)_ = 26.856, *p* < 0.0001]. This prolongation was most evident for subjects in the youngest group (group C: 5–14 years), but was also present in the other age groups [Figure 4A; effect of age group: *F*_(3, 570)_ = 16.278, *p* < 0.0001; saccade to target X age group: *F*_(3, 570)_ = 1.65, *p* = 0.1793; *post-hoc* analysis: group C: *p* < 0.0001; Y: *p* = 0.0216; M: *p* = 0.0346; E: *p* = 0.0007].

**Figure 5:**
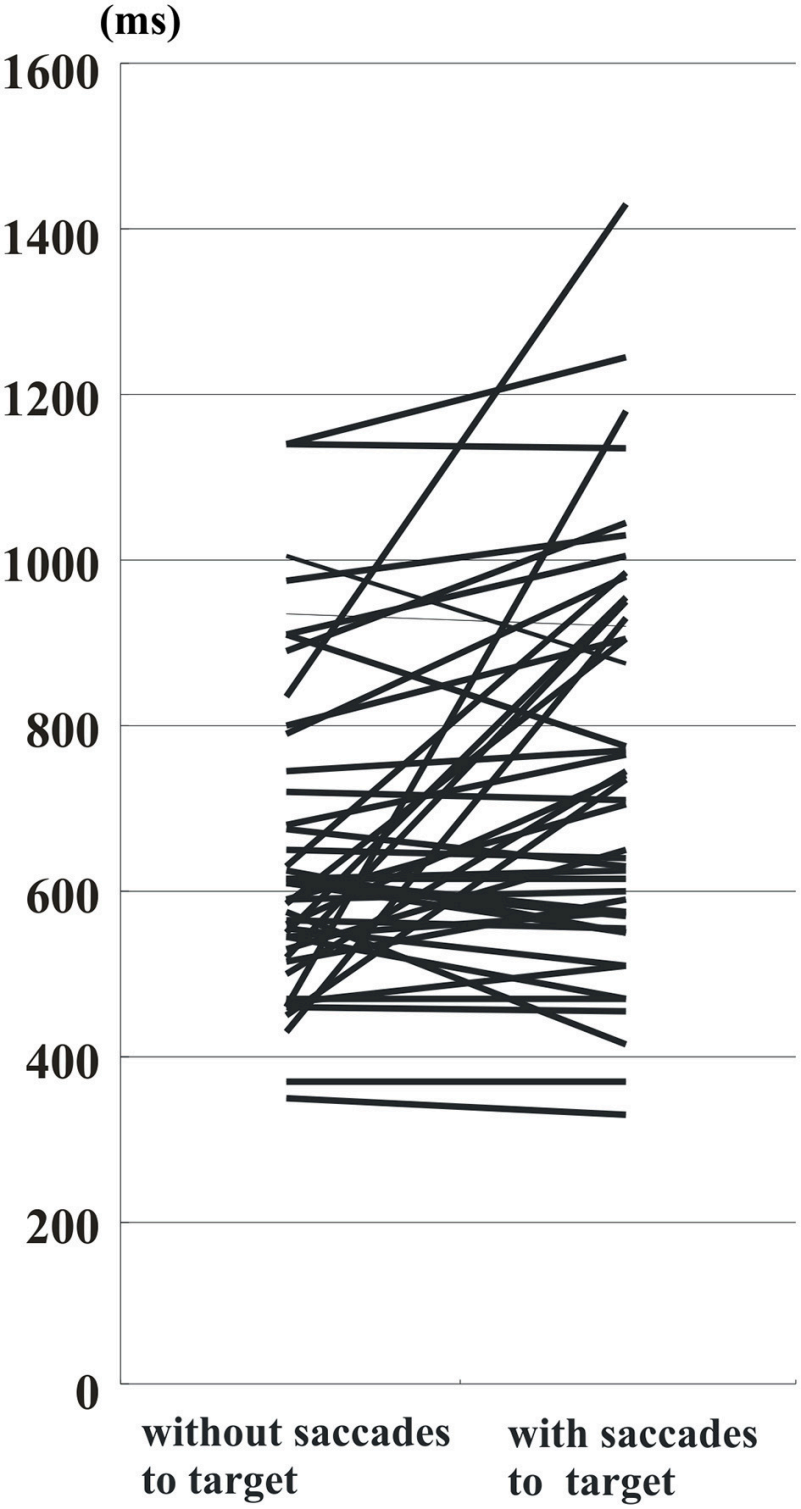
**RT of PD patients in trials with and without saccades to target**. Conventions as in Figure 3. Data from the same patients are connected for trials without saccades to target (left side of figure) and with saccades to target (right side of figure). Note that RT tends to become prolonged after saccades to target in most of the subjects.

Similarly, the histogram of hand RT in PD patients showed a shift toward longer RT when patients made saccades to target (blue bars) than when they did not (yellow bars) (Figure 4B). The delay after saccades to target was also compared on an individual subject basis (Figure 5). On average, without saccades to target (650.1 ± 28.7 ms), hand RT was significantly shorter than when subjects made a saccade to target (747.4 ± 37.0 ms), and this difference reached significance (paired Student's *t*-test: *p* = 0.00166). Therefore, overall, PD patients showed a slower motor response after they made a saccade to target than when they did not.

## Discussion

We investigated the effect of inadvertent saccade intrusion on the initiation of subsequent oculomotor/motor actions in normal subjects as well as PD patients. Both MGS and hand RT tasks required the subjects to keep gazing at the central fixation point until an imperative signal prompted them to initiate a quick voluntary oculomotor or motor response. The difference between these two tasks is that the MGS task required subjects to perform a voluntary oculomotor response, while the hand RT task required a hand motor reaction. In the MGS task, an unwanted saccade intrusion could occur some time before the voluntary oculomotor action. In the hand RT task, an unwanted saccade intrusion might or might not co-occur with the hand motor action in close temporal proximity. In both of these situations, the latencies of motor and oculomotor reactions increased as a result of a preceding saccade intrusion in normal subjects.

### Delay in voluntary saccades after inadvertent saccade intrusions

In normal subjects, delay in voluntary saccades after inadvertent saccade intrusions was noted in all age ranges, although it was most evident for the youngest group (5–14 years). Similarly, in 17 of the 49 PD patients who could perform MGS with a relatively normal latency and normal success rate, the latency of subsequent MGS was delayed after inadvertent saccades were made, just as observed in normal subjects (Figure 3A).

One possible explanation of the longer MGS latency in these subjects after saccades to cue may be related to attentional control. The term “inhibition of return” (IOR) refers to a phenomenon in which subjects are temporarily slower to respond to stimuli that are presented at previously cued locations (Posner, 1980; Posner and Cohen, 1984; Posner et al., 1985; Klein, 1998, 2000). Thus, IOR implies a relative suppression of processing of stimuli, such as responding to a visual stimulus that had recently been the focus of attention. Once subjects have made a saccade to cue in the MGS task, a locus of suppression may arise in the activated corresponding locus of the superior colliculus (SC; Dorris et al., 1999, 2002), and this may make the latency of voluntary saccades (in this case, MGS) to the same location slower to initiate. In the MGS task, saccades to cue occurred mostly 100–200 ms after the presentation of the cue, and the imperative signal (i.e., extinction of the central fixation point) occurred 1.6–2.4 s after the cue presentation. The entire time course is compatible with the occurrence of IOR. Dorris et al. (2002) suggested that the reduced activity of the SC accompanying IOR does not take place in the SC itself, but actually reflects a signal reduction that has taken place upstream of the SC, such as in the cortical oculomotor regions, including the frontal eye fields (FEFs), supplementary eye fields, and the posterior parietal cortex and subcortical areas, including the substantia nigra. Among these, the parietal cortex, which is engaged in attentional control (Corbetta and Shulman, 2002, for review), is considered one of the most important neural structures responsible for IOR (Dorris et al., 2002). In other words, once the subjects' attention is distracted by the cue in the MGS task, they become slower for some time in performing the reaction required for the upcoming task.

A second possible explanation for MGS delay after saccade intrusion, which is not mutually exclusive with the preceding explanation, is related to task set switching or modulation of neural activity by preceding trial history. It is known that behavior slows down when switching between controlled and automatic behavior (Cherkasova et al., 2002; Vernet et al., 2009; Weiler and Heath, 2012, 2014; Hodgson et al., 2013, 2015). The effect of saccade intrusions on subsequent voluntary saccades may reflect a similar switching cost: saccades to cue can be considered to represent visually guided saccades to the cue, whereas the ensuing MGS can be considered voluntarily controlled saccades. Once saccades to cue (an automatic saccade) have been made, the ensuing MGS (a controlled saccade) would have an increased latency.

A similar increase of saccade latency after task switching is observed in switching between prosaccades and antisaccades: when the previous trial is an antisaccade, the latencies of both prosaccades and antisaccades (one version of controlled saccades) are prolonged. Functional MRI has shown that antisaccades are associated with reduced FEF activity relative to those preceded by prosaccades (Manoach et al., 2007). This suggests that neural activity is modulated by trial history, consistent with a rapid, dynamic form of learning. The activity of FEF may be modulated in a similar manner, when forthcoming voluntary saccades (e.g., MGS) are preceded by saccade intrusions (reflexive saccades).

Notably, behavioral data suggest that microsaccades delay subsequent behavior, including the subsequent execution of saccades (Rolfs et al., 2006, 2008). This has been explained by the temporary suppression of the SC cells coding the target location of saccade after microsaccades, leading to delay of subsequent saccade execution (Rolfs and Ohl, 2011). Furthermore, saccades and microsaccades have been shown to share similar generative mechanisms with saccades of larger amplitude, including a causal role of the SC, even in neurological patients (Hafed et al., 2009; Otero-Millan et al., 2013). Thus, the third possible account may be that the saccade intrusions in the present study, in the range of 0.1–43.4° (median 4.7°), may have delayed the subsequent voluntary saccade or voluntary action by a similar mechanism for microsaccades. In the present study, the timing of saccade intrusion preceded the oculomotor reaction in MGS and hand reaction in the RT task by about 1600–2400 ms. Actually, this interval is longer than the interval with which microsaccades precede subsequent saccades when the former affects the latency of the latter (up to 800 ms according to Rolfs et al., 2006, 2008). In addition, the amplitude of microsaccades is known to affect the amount of delay induced (Rolfs et al., 2008); microsaccades with larger amplitudes are followed by longer response latencies. If we postulate a similar mechanism for the delay of voluntary oculomotor and motor action after saccade intrusions, saccade intrusions of larger amplitudes would have induced a larger delay in these actions in a similar manner as the microsaccades. In the present study, we did not observe this relationship.

However, the overall delay in MGS latency after saccades to cue in PD patients was shorter compared with normal subjects (Figures 2A,B). In the 17 PD patients who could perform MGS within the normal latency range, MGS latency was almost unchanged or slightly shorter after saccades to cue. These PD patients were relatively old, similar in age range to elderly normal subjects. The smaller change in MGS latency with and without saccades to cue may suggest milder modulation of the oculomotor system in elderly subjects compared with younger subjects (Figure 2A). Furthermore, in these PD patients, the output of oculomotor system for volitional saccades, especially the SC, may be excessively inhibited by overactive basal ganglia output, to such an extent that it can no longer undergo normal modulation (Terao et al., 2013c).

In the other 19 PD patients, who had difficulty in performing MGS within the cutoff time limit (670 ms after the offset of central fixation point), saccade latency was frequently shorter after saccades to cue than when no such saccade intrusions occurred (Figure 3B). These PD patients were unable to initiate internally guided MGS in the absence of a visual target, but were able to make a visually guided saccade (externally guided) to the second presentation of the target. Thus, for making these visually guided saccades, they actually did not have to switch between automatic and controlled behavior. For these patients, it was impossible to explain the change in MGS latency after saccades to cue by IOR, since IOR would actually slow saccade latency rather than to shorten it.

It is possible that some PD patients would not have been able to initiate MGS because the interval between the fixation point and the target presentation for the second time was relatively short (600 ms); if the interval was longer, even PD patients could have generated memory guided saccades in all trials. However, the histogram in Figure 11 of our previous paper (Terao et al., 2011a) shows that, even in PD patients with advanced stages of H–Y stage 3–4, PD patients would have been able to perform internally guided saccades within 600 ms after the fixation offset in over 90% of the trials in most patients who were able to initiate voluntary saccades at all. After 670 ms, the tendency to make visually guided saccades rose rapidly. On the other hand, after 670 ms, the tendency to make visually guided saccades rose rapidly. As a result, for PD patients who could perform MGS with a normal latency at all, majority of the saccades were internally guided, and for PD patients who could not, most of the saccades were visually guided, and there were relatively little “mixing” of these two types. Although we used a period of 600 ms, we would have observed the same trend even when we used a longer period between the fixation point and the target presentation for the second time.

Why was the latency of subsequent MGS shorter after saccades to cue in these PD patients? The SC receives input from the basal ganglia for initiating and inhibiting saccades. Various studies have shown that in PD, pathological rhythmicity develops in the basal ganglia (BG) circuit and also in the SC which is receiving this input, and this jeopardizes the oculomotor processing both for initiating and suppressing saccades (Bergman et al., 1998; Brown, 2003).

The basal ganglia model of Nambu et al. (2000) and Nambu (2004) may be invoked to explain the possible modulation of SC activity associated with saccade initiation. According to this model, when a movement (including saccades) is about to be initiated by cortical mechanisms, a corollary signal is sent through the hyperdirect pathway from the cortex to the subthalamic nucleus to first inhibit large areas of the thalamus and cerebral cortex [for eye movements, the substantia nigra parts reticalata (SNr) and the SC] that are related to both the selected motor program and other competing programs. Subsequently, another corollary signal is sent through the direct cortico-striato-pallidal pathway to disinhibit the targets of the direct pathway, and to ensure activation of only the selected motor program. Finally, a third corollary signal is sent through the indirect cortico-striato-external pallido-subthalamo-internal pallidal pathway to strongly inhibit the targets of this third pathway. In normal subjects, the sequential process of inhibition-facilitation-inhibition is thought to ensure that only the selected motor program is initiated, executed and terminated at the appropriate times, whereas other competing programs are canceled.

When a visual cue is present and the subjects have made a saccade to cue, the BG circuit and its output to the SC would be broadly inhibited. Normally, such modulation may work to “reset” the entire oculomotor system, and would slow the initiation of subsequent saccades, as was observed in normal subjects. In PD patients, however, the abnormal rhythmicity in the BG and SC would be swept away by the resetting that occurred after inadvertent saccades. Consequently, subsequent saccades would be processed more quickly and initiated with a shorter latency than without saccades to cue.

In summary, saccade intrusion did not largely increase MGS latency in PD patients who could perform MGS with a relatively normal latency. In contrast, PD patients who were unable to initiate MGS within the normal range, showed slightly shorter MGS latency after the occurrence of saccade intrusions.

### Delay in voluntary motor reaction after saccade intrusions

In the hand RT task, we also found that inadvertent saccades made to the visual target (saccades to target) delayed the initiation of subsequent hand movements in response to the same cue in both normal subjects and PD patients. In normal subjects, this delay was most pronounced in the youngest subject group and tended to grow less evident with age. In PD patients, the overall RT distribution was shifted toward a longer range relative to normal subjects both with and without saccades to target, but also showed a delay after saccades to target (Figure 4).

The RT task involved two possible effectors, the eyes and the hand, although the instruction was to respond with the hand but not with the eyes. Our results indicate that responding with the non-instructed eyes delayed the initiation of the instructed hand motor reaction. Although the exact mechanism for this delay is unclear, one possible explanation may be the shared initiation process between different effectors, involving the right posterior superior temporal lobe and left ventral premotor cortex (Kansaku et al., 2004). According to their model, the initiation process can be shared among different types of sensory stimuli and output movements, and only after specifying the effector can a movement be initiated. Once one of the effectors has been selected to perform an action—for example, when a gaze movement has been made before the hand has reacted, subsequent motor action for other effectors may be inhibited—areas more directly involved in generating hand movements (e.g., the motor cortex) may be inhibited by non-preferred effector types (in this case, eye movements). In our hand RT task, saccades to cue would thus disrupt the second stage of processing, and thus the hand reaction was delayed.

Interestingly, the left ventral premotor cortex, which is thought to play an important role in the shared initiation process, is almost identical to the area that forms part of a wider frontal network mediating inhibitory control over stimulus-elicited eye movements, i.e., saccades to cue and saccades to target in the present study (Hodgson et al., 2007), whereas the homologous area in the right ventral premotor cortex is involved in rule task switching (Hodgson et al., 2007, 2013, 2015).

Another possible explanation of the delayed RT after making a saccade to target may be provided by deficient attentional control, i.e., IOR as discussed above (Posner, 1980; Posner and Cohen, 1984; Posner et al., 1985; Klein, 1998, 2000). Once the subject makes a saccade to target, his/her attention may be temporarily distracted from the task at hand. The RT task in this study required the subjects not to make saccades to the same location. Thus, after a saccade intrusion, a locus of suppression may arise at the same region of the SC to terminate the saccade and bring the eyes back to their original location (see Dorris et al., 1999, 2002). Therefore, after making a saccade to cue, hand RT in response to visual stimuli appearing at the same location would be longer than without a saccade to cue. Indeed, similar paradigms have been shown to affect not only saccadic but also manual RTs, which suggests that this effect may occur regardless of the effector used for motor action (Dorris et al., 1999). Thus, saccade intrusions may delay the initiation of subsequent voluntary motor actions.

In normal subjects, RT was increased by saccade intrusions, especially in the youngest subject group. In children, the control of oculomotor and motor systems, somewhere within the shared and segregated processes of motor initiation, may not have developed sufficiently to achieve independence, as seen in adults. If the subject makes a saccade to target, not only subsequent voluntary saccades but also subsequent motor actions would be delayed, since both the neural systems required for oculomotor and motor control become inhibited due to the lack of selectivity. However, as the independent and selective control between the hand and the eyes develop with age, RT may become less affected by saccades to target, although an increase in RT after saccade intrusion was still observed in adult subjects (Figure 5).

Since PD patients are impaired in suppressing reactive saccades to a suddenly appearing visual stimuli (Terao et al., 2011a, 2013c), their tendency for overt and covert attentional shift may be exaggerated more than in normal subjects, leading to slowed initiation of subsequent voluntary action. We thus expected that the delay of hand RT after saccades to target would be larger in PD patients than in normal subjects. Indeed, the delay was smaller for PD patients. The reason for the smaller effect of saccades to target in PD patients may be that the baseline RT in PD patients is relatively long compared with young subjects, which makes their RT less affected by saccades to target.

### Clinical implications

As mentioned in the Introduction, clinical treatment to restore gaze control, especially inhibitory control of gaze, can be expected to substantially ameliorate the motor delays associated with saccade intrusion. While deep brain stimulation of the subthalamic nucleus (STN DBS) in PD patients changes the small amplitude and multiple electromyographic bursts of limb movements into a large single-step movement with larger EMG size (Kumru et al., 2004; Sauleau et al., 2008), it is also known to be effective in suppressing saccades to cue (Yugeta et al., 2010). Thus, DBS would be important not only in suppressing prepotent but unnecessary actions, but also in preventing motor delays induced by inadvertent reactive eye movements.

On the other hand, some saccade intrusions (saccades made to target presented for the second time in the MGS task) can make subsequent saccades faster to perform. PD patients with prolonged MGS latency were able to make saccades more quickly after making saccades to cue (Figure 3B). However, as noted above, these saccades were not voluntary saccades (MGS) made in the absence of visual targets, but were actually visually triggered saccades made in response to the sudden appearance of visual targets. Furthermore, even these “faster” responses had a longer latency compared with normally performed voluntary saccades (Figures 3A,B). Deficient modulation of the BG prevents these PD patients from performing voluntary saccades with minimal latency, and they have to adjust for this by making visually guided saccades in an awkward manner. Similar strategic changes may be adopted by patients in the clinical setting, such as the paradoxical gait. However, this coping strategy would fail in the absence of visual triggers.

The phenomena we observed may have a possible link with the behavior of patients with attention-deficit hyperactive disorder and obsessive-compulsive disorder, who show impaired impulse control and delayed psychomotor development and whose underlying pathophysiology may be related to abnormal limbic-brainstem interaction. These patients exhibit reduced ability to inhibit prepotent responses, and they also show a slowing of psychomotor function in an attempt to compensate for inhibitory deficits by slowing reaction times to better inhibit reflexive responses (Mosconi et al., 2009; Bueno et al., 2014; Schmitt et al., 2014; Petrovic and Castellanos, 2016).

## Author contributions

Conceived and designed the experiments: YT, HF, and YU. Performed the experiments: YT, HF, ST, and SI. Analyzed the data: YT, HF, ST, and SI. Contributed reagents/materials/analysis tools: YT, HF, ST, and SI. Wrote the paper: YT, HF, and YU.

## Funding

YT was supported by a Research Project Grant-in-Aid for Scientific Research from the Ministry of Education, Culture, Sports, Science, and Technology of Japan [16K09709, 16H01497]. YU was supported by a Research Project Grant-in-Aid for Scientific Research from the Ministry of Education, Culture, Sports, Science and Technology of Japan [No. 25293206, No. 22390181, 15H05881, 16H05322]; by grants from the Research Committee on the Best rTMS Treatment of Parkinson's Disease from the Ministry of Health and Welfare of Japan; and by the Research Committee on Dystonia of the Ministry of Health and Welfare of Japan.

### Conflict of interest statement

The authors declare that the research was conducted in the absence of any commercial or financial relationships that could be construed as a potential conflict of interest. The reviewer HS and handling Editor declared their shared affiliation, and the handling Editor states that the process nevertheless met the standards of a fair and objective review.
